# Protocol for the EMPHASIS study; epigenetic mechanisms linking maternal pre-conceptional nutrition and children’s health in India and Sub-Saharan Africa

**DOI:** 10.1186/s40795-017-0200-0

**Published:** 2017-10-30

**Authors:** Giriraj R. Chandak, Matt J. Silver, Ayden Saffari, Karen A. Lillycrop, Smeeta Shrestha, Sirazul Ameen Sahariah, Chiara Di Gravio, Gail Goldberg, Ashutosh Singh Tomar, Modupeh Betts, Sara Sajjadi, Lena Acolatse, Philip James, Prachand Issarapu, Kalyanaraman Kumaran, Ramesh D. Potdar, Andrew M. Prentice, Caroline H. D. Fall, the EMPHASIS study group, Lena Acolatse, Meraj Ahmed, Modupeh Betts, Giriraj R. Chandak, Harsha Chopra, Cyrus Cooper, Momodou K. Darboe, Chiara Di Gravio, Caroline H. D. Fall, Meera Gandhi, Gail R. Goldberg, Prachand Issarapu, Philip James, Ramatoulie Janha, Landing M. A. Jarjou, Lovejeet Kaur, Sarah H. Kehoe, Kalyanaraman Kumaran, Karen A. Lillycrop, Mohammed Ngum, Suraj S. Nongmaithem, Stephen Owens, Ramesh D. Potdar, Andrew M. Prentice, Ann Prentice, Tallapragada Divya Sri Priyanka, Ayden Saffari, Sirazul Ameen Sahariah, Sara Sajjadi, Harshad Sane, Smeeta Shrestha, Matt J. Silver, Ashutosh Singh Tomar, Kate A. Ward, Dilip Kumar Yadav, Chittaranjan S. Yajnik

**Affiliations:** 1 CSIR-Centre for Cellular and Molecular Biology, Hyderabad, India; 2 MRC Unit The Gambia and MRC International Nutrition Group, London School of Hygiene and Tropical Medicine, London, UK; 3 University of Southampton, Southampton, UK; 4 Centre for the Study of Social Change, Mumbai, India; 5 MRC Lifecourse Epidemiology Unit, University of Southampton, Southampton General Hospital, Tremona Road, Southampton, SO16 6YD UK; 6 MRC Elsie Widdowson Laboratory, Cambridge, UK; 7 MRC Unit, The Gambia, Serrekunda, Gambia; 8 MRC Lifecourse Epidemiology Unit, University of Southampton, UK and CSI Holdsworth Memorial Hospital, Mysore, India; 9 MRC Unit, The Gambia and MRC International Nutrition Group, London School of Hygiene and Tropical Medicine, London, UK

**Keywords:** Pre- and peri-conceptional nutrition, Epigenetics, DNA methylation, Children, Growth, Body composition, Bone density, Non-communicable disease (NCD) risk markers, Cognitive function, Developmental origins of health and disease (DOHaD)

## Abstract

**Background:**

Animal studies have shown that nutritional exposures during pregnancy can modify epigenetic marks regulating fetal development and susceptibility to later disease, providing a plausible mechanism to explain the developmental origins of health and disease. Human observational studies have shown that maternal peri-conceptional diet predicts DNA methylation in offspring. However, a causal pathway from maternal diet, through changes in DNA methylation, to later health outcomes has yet to be established. The EMPHASIS study (Epigenetic Mechanisms linking Pre-conceptional nutrition and Health Assessed in India and Sub-Saharan Africa, ISRCTN14266771) will investigate epigenetically mediated links between peri-conceptional nutrition and health-related outcomes in children whose mothers participated in two randomized controlled trials of micronutrient supplementation before and during pregnancy.

**Methods:**

The original trials were the Mumbai Maternal Nutrition Project (MMNP, ISRCTN62811278) in which Indian women were offered a daily snack made from micronutrient-rich foods or low-micronutrient foods (controls), and the Peri-conceptional Multiple Micronutrient Supplementation Trial (PMMST, ISRCTN13687662) in rural Gambia, in which women were offered a daily multiple micronutrient (UNIMMAP) tablet or placebo. In the EMPHASIS study, DNA methylation will be analysed in the children of these women (~1100 children aged 5–7 y in MMNP and 298 children aged 7–9 y in PMMST). Cohort-specific and cross-cohort effects will be explored. Differences in DNA methylation between allocation groups will be identified using the Illumina Infinium MethylationEPIC array, and by pyrosequencing top hits and selected candidate loci. Associations will be analysed between DNA methylation and health-related phenotypic outcomes, including size at birth, and children’s post-natal growth, body composition, skeletal development, cardio-metabolic risk markers (blood pressure, serum lipids, plasma glucose and insulin) and cognitive function. Pathways analysis will be used to test for enrichment of nutrition-sensitive loci in biological pathways. Causal mechanisms for nutrition-methylation-phenotype associations will be explored using Mendelian Randomization. Associations between methylation unrelated to supplementation and phenotypes will also be analysed.

**Conclusion:**

The study will increase understanding of the epigenetic mechanisms underpinning the long-term impact of maternal nutrition on offspring health. It will potentially lead to better nutritional interventions for mothers preparing for pregnancy, and to identification of early life biomarkers of later disease risk.

## Background

EMPHASIS (Epigenetic Mechanisms linking Pre-conceptional nutrition and Health Assessed in India and Sub-Saharan Africa, www.emphasisstudy.org) is a collaboration between investigators in the UK, India and The Gambia designed to profile genome-wide DNA methylation in children whose mothers participated in two pre- and peri-conceptional micronutrient supplementation trials (the Mumbai Maternal Nutrition Project in India, MMNP [1] and the Peri-conceptional Multiple Micronutrient Supplementation Trial, PMMST, in rural Gambia [2]). The main objectives of the study are to identify methylation differences associated with the interventions and correlate these with health-related phenotypes in the children, including size at birth, post-natal growth, and childhood body composition, skeletal health, cardiometabolic risk markers and cognitive function. We hypothesise that maternal nutritional supplementation around the time of conception will result in altered DNA methylation profiles in the children, and that the distinct methylation patterns identified will show potentially causal associations with phenotypic characteristics in the children. We further expect to identify methylation differences unrelated to supplementation that are associated with the measured phenotypes.

### Context

Poor quality diets and the resulting micronutrient deficiencies are major public health problems in low-and-middle-income countries (LMICs). In pregnant women they impair fetal development, and recent evidence suggests that they are also associated with longer term health problems in the offspring including stunting [3], impaired neurodevelopment [4] and, through ‘metabolic programming’, with increased vulnerability to adult non-communicable chronic diseases (NCDs) such as obesity, type 2 diabetes, cardiovascular disease and osteoporosis [5–7].

Long-term effects of fetal nutrition on later health would require mechanisms by which a ‘memory’ of the early environment is retained into later life and influences metabolism. Epigenetic signatures, including patterns of DNA methylation that are modifiable by environmental exposures, are leading candidate mechanisms [8, 9]. DNA methylation is a mitotically heritable epigenetic mark that plays a key role in the transcriptional regulation of cellular processes, including cell differentiation, genomic imprinting and X-chromosome inactivation. DNA methylation depends on the supply of methyl groups through the 1-carbon pathway, which requires vitamins B2, B6, B12, folate, methionine, choline and betaine, and amino acids serine and glycine, for normal function. The peri-conceptional period is a critical window when the process of establishing methylation marks is sensitive to nutrition [8, 9].

The initial ‘proof of principle’ of nutritional programming mediated by changes in DNA methylation came from the Agouti mouse model, in which natural variation in methylation at the Agouti locus influences coat colour, adult adiposity and glucose tolerance [8]. This locus is a metastable epiallele (ME), a genomic region characterised by inter-individual variation in methylation patterns that are established in the early embryo before gastrulation, and are therefore highly correlated across tissues derived from all three germ layers. Feeding pregnant dams ‘methyl donor’ nutrients (vitamin B12, folic acid, betaine and choline) increased methylation of the agouti locus and reduced agouti gene expression, leading to fewer obese yellow offspring and more lean brown offspring, characteristics that persisted into adult life [8]. Dietary manipulations in pregnancy affect the methylation and expression of offspring genes other than MEs. For example, in rats, maternal protein-restriction reduces methylation of offspring peroxisome proliferator activated receptor 1 alpha (*PPARα*) and glucocorticoid and angiotensin receptor genes [10]. Maternal folic acid supplementation prevents both the methylation and phenotypic effects (e.g. high blood pressure) induced by maternal protein restriction in the offspring.

There is evidence in humans that epigenetic changes induced by the nutritional environment in early life alter later phenotype, including body composition and cardiometabolic health. For example, DNA methylation at a number of loci (insulin-like growth factor 2 (*IGF2*), retinoid X receptor alpha (RXRA), endothelial nitric oxide synthase (*eNOS*), *PGC1α*, and cyclin-dependent kinase inhibitor 2 (*CDKN2a*) genes in cord tissue, cord blood or children’s leucocytes is associated with adiposity in later childhood [11–14]. Studies in The Gambia, where there is marked seasonal variation in maternal nutrition, have shown that season of conception is related to DNA methylation of human infant MEs [15], and that methylation is predicted by elements of the mother’s methyl donor metabolome at conception [16]. One implicated gene is the maternally imprinted tumour suppressor and immune function regulator vault RNA2–1 (*VTRNA2–1*) [17], making it a promising candidate for exploring mechanisms linking season of conception and infectious disease mortality in Gambians [18]. A methylation variant affecting expression of the pro-opio-melanocortin (*POMC*) gene has also been associated with child and adult obesity. This locus is an ME, and in Gambian infants methylation is associated with season of conception and maternal 1-carbon metabolites at conception [19].

Evidence for epigenetic programming in humans has hitherto relied mainly on observational studies. Randomised trials of peri-conceptional maternal nutritional interventions with follow-up of the children represent a stronger study design in which to examine effects on DNA methylation and health-related phenotypes, reducing the problems of confounding and bias that affect observational studies, thus providing stronger evidence of causality. The EMPHASIS study will be an important step towards understanding mechanisms underpinning the developmental origins of health and disease (DOHaD), identifying biomarkers of early life exposures associated with later disease risk, and designing more effective nutritional interventions for mothers preparing for pregnancy.

## Design and methods

EMPHASIS is a follow-up study of two cohorts of children born to mothers who took part in separate randomized controlled trials of nutritional supplementation before and during pregnancy.

### The original trials and the cohorts of children

#### Mumbai maternal nutrition project

MMNP (also known as Project SARAS [‘excellent’]; ISRCTN62811278) was a non-blinded individually randomized trial among Indian women living in Mumbai slums (2006–2012) [1]. The intervention was a daily snack, eaten in addition to normal diet, made from naturally micronutrient-rich local foods (green leafy vegetables, fruit and milk). Control snacks contained foods of low micronutrient content (e.g. potato, onion). Intervention snacks contained 10–23% of the WHO Reference Nutrient Intake (RNI) for β-carotene, vitamins B2 and B12, folate, calcium and iron, and 0.7 MJ of energy and 6 g of protein, compared with 0–7% RNI for the micronutrients, 0.4 MJ of energy and 2 g of protein in control snacks. At recruitment, non-pregnant women had detailed anthropometry, and data were collected on socio-economic status (Standard of Living Index [20]) and habitual diet by food frequency questionnaire. They received either intervention or control snacks; intake was supervised and recorded daily. Women who became pregnant continued supplementation until delivery, and were supplied with routine iron (100 mg) and folic acid (500 μg) supplements as per Indian government recommendations. Fetal biometry was recorded three times during pregnancy (at approximately 10, 20 and 29 weeks gestation, estimated from last menstrual period date and ultrasound measures) [21]. Plasma folate and vitamin B12 concentrations were measured in early pregnancy (~10 weeks gestation). An oral glucose tolerance test (WHO 1999 protocol) was performed at 28–32 weeks gestation [22]. Main outcomes were newborn anthropometry and gestational age at delivery. Of 6513 women recruited, 2291 became pregnant, leading to 1962 live singleton deliveries.

In the intention to treat analysis, there were no differences in birth weight or other newborn measurements between allocation groups [1]. In the per protocol analysis, limited to women who started supplementation at least 3 months before conception, a period that was considered long enough to achieve the maximal effect on maternal nutritional status, birth weight increased by a mean 48 g (*p* = 0.05). In both analyses there was an interaction between maternal BMI and the intervention, with a larger birth weight effect in mothers of BMI >18.5 kg/m^2^ (intention to treat: +63 g [95%CI 11, 115]; per protocol: +96 g [95%CI 35, 154]; p for interaction 0.001). The intervention reduced the prevalence of gestational diabetes (intention to treat: 7.3% compared with 12.4% in controls; OR: 0.56; 95% CI: 0.36, 0.86; *P* = 0.008) [22]. It had no effect on fetal size assessed using standard ultrasound measures [21].

**Fig. 1:**
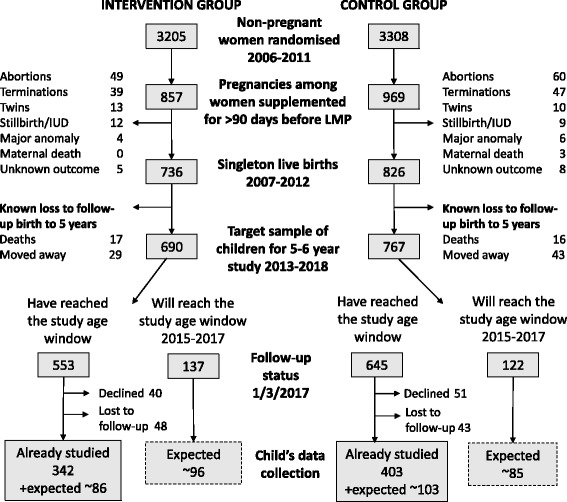
Flow diagram of the MMNP trial in Mumbai, India and children’s follow-up (SARAS KIDS)

The children of mothers who participated in MMNP are currently (2013–2018) being studied at 5–7 years of age (“SARAS KIDS” study) to measure anthropometry, body composition, skeletal development, cardio-metabolic risk markers and cognitive function (Fig. 1, Tables 1 and 2). Venous blood samples and buccal swabs are collected for DNA and RNA, and are stored in -80 °C freezers until transportation in batches to the laboratory on dry ice. The DNA samples and phenotype data will be used for the EMPHASIS study, in which we will limit the sample to the 1562 children born to mothers in the per protocol group. Data collection will be completed by the end of January 2018.

**Table 1:** Maternal, newborn and child characteristics for the children in India and The Gambia who have participated in the EMPHASIS study

	MMNP, India		PMMST, The Gambia	
Mothers				
N	1562		376	
Age at conception (y)^a^	24	(21, 27)	29	(29, 35)
Pre-pregnant BMI (kg/m^2^)^a^	19.7	(17.8, 22.4)	20.8	(19.3, 22.9)
Pre-pregnant height (cm)	151.4	(5.4)	161.0	(5.5)
Primiparous	489	(31)	26	(7)
Live singleton newborns				
N	1562		376	
Birth weight (g)	2606	(400)	3035	(417)
Birth length (cm)	47.6	(2.4)	49.8	(2.4)
SGA (N(%))	732	(47)	46	(12)
Pre-term (N(%))	205	(13)	33	(9)
Children at the time of DNA collection				
N	709^b^		298	
N with adequate DNA sample	698		293	
Age (y)^a^	5.8	(5.6, 6.0)	9.0	(8.6, 9.2)
Weight (kg)	16.2	(2.5)	23.0	(3.2)
Weight SD score (WHO/CDC)	−1.7	(1.1)	−1.4	(0.9)
Height (cm)	109.6	(4.9)	127.7	(5.4)
Height SD score (WHO/CDC)	−1.0	(1.0)	−0.7	(0.8)
BMI (kg/m^2^)	13.4	(1.4)	14.1	(1.2)
BMI SD score (WHO/CDC)	−1.6	(1.1)	−1.4	(1.0)

Abbreviations: *BMI* body mass index, *SGA* small for gestational age, *SD score*: standard deviation score, *WHO* World Health Organization, *CDC* Centers for Disease Control and Prevention

^a^ Median (IQR); other figures shown are mean (SD), or N (%) where indicated

^b^ Data collection is ongoing in the Mumbai study; figure given is up to 28th February 2017

**Table 2:** Data collected among the Indian and Gambian children

Measurements	SARAS KIDS children India	PMMST children The Gambia
Anthropometry	Weight, standing and sitting height; mid-upper-arm circumference; head, chest and abdominal circumferences, skinfolds (triceps, biceps, sub-scapular and supra-iliac) using standardised protocols.	
Blood pressure	After 5 min seated at rest. Mean of 3 readings of systolic and diastolic pressure from left upper arm. Instrument: OMRON HEM7080.	
Biochemistry	Plasma glucose concentrations after an overnight fast of ≥8 h and 30 and 120 min after a 1.75 g/kg oral anhydrous glucose load. Measured by standard enzymic methods on an autoanalyzer (India: Hitachi 902, Roche Diagnostics, Mannheim, Germany; The Gambia: Cobas Integra 400 Plus Biochemistry Analyzer, Roche Diagnostics). Plasma insulin fasting and 30 mins after the glucose load Measured by a Mercodia ELISA assay on a Victor 2 analyzer, Turku, Finland, in India and by an SM-chemiluminescence method on an Architect i1000 Plus analyzer, Abbott in The Gambia. Plasma fasting total, LDL- and HDL-cholesterol and triglycerides by standard enzymic methods (India: Hitachi 902; The Gambia: Cobas Integra 400 Plus).	
Body composition	Total and regional (arms, legs, trunk, android and gynoid) fat mass, lean mass and body fat % using dual-energy x-ray absorptiometry (DXA, Lunar Prodigy in India and Lunar iDXA in The Gambia, GE Medical Systems, GE Lunar Corporation, Madison USA).	
Skeletal development	Bone area (BA), bone mineral content (BMC), and bone mineral density (BMD) measured using dual-energy x-ray absorptiometry (DXA; Lunar Prodigy in India and Lunar iDXA in The Gambia).	
	–	Tibial total and trabecular volumetric bone mineral density (vBMD), and BA; and diaphysial BA, cortical area, thickness, BMC, cortical vBMD and strength (cross-sectional moment of inertia) measured using peripheral quantitative computed tomography (pQCT; Stratec XCT 2000, Stratec Ltd., Pforzheim, Germany).
Cognitive function	Three core tests from the Kaufman Assessment Battery for children, 2nd edition, 2004 (KABC II) – Atlantis (learning ability, long-term storage and retrieval, associative memory); Word order (memory span, short-term memory, working memory); Pattern reasoning (reasoning ability, induction, deduction, fluid reasoning) [38]. Additional tests from the Wechsler Intelligence Scale for Children (WISC): Kohs block design (visuo-spatial problem-solving, visual perception and organisation); Coding-Wisc III (visual-motor processing speed and co-ordination, short-term memory, visual perception, visual scanning, cognitive flexibility, attention); Verbal fluency (a) animals, (b) names (broad retrieval ability, speed and flexibility of verbal thought processes, neuropsychological test of language production) [39–41].	
DNA and RNA	Whole blood collected into EDTA tubes and DNA isolated using Qiagen DNA Blood Midi Kit. DNA methylation measured in a single laboratory (CSIR-Centre for Cellular and Molecular Biology, Hyderabad, India) using (genome wide) Illumina Infinium MethylationEPIC arrays and (locus-specific) bisulfite sequencing on Pyromark96 (see main text for more details). High-resolution genotype data generated using Illumina Global Screening Array. Buccal DNA obtained using Isohelix buccal swabs in India and Mawi iSwab kits in The Gambia. Whole blood samples collected into Paxgene tubes for later RNA isolation.	
Full blood count	Hemoglobin, red cell count and indices, differential white blood cell count (India: Pentra XL Retic analyzer, Horiba Medical, Montpellier, France; The Gambia: Medonic hematology analyzer, Spanga, Sweden).	

#### West kiang Peri-conceptional multiple micronutrient supplementation trial

**Fig. 2:**
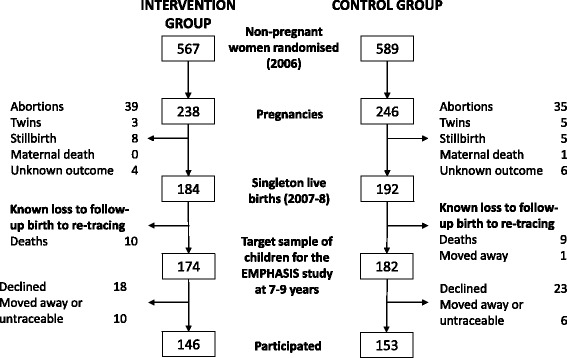
Flow diagram of the PMMST trial in The Gambia, and the children’s follow-up

PMMST (ISRCTN13687662) was a double-blind individually randomized trial among women living in rural West Kiang, The Gambia (2006–2008) [2] (Fig. 2). The intervention was a daily multiple micronutrient tablet (UNIMMAP) providing the RNI of vitamins A, B1, B2, niacin, B6, folic acid, B12, C, D and E and iron, zinc, copper, selenium and iodine [23]. Control women received matching placebo tablets. At recruitment, non-pregnant women had anthropometry. Compliance with supplementation was assessed by fortnightly tablet counts. Women stopped the supplement when they became pregnant, confirmed either by pregnancy test or by ultrasound at approximately 12 weeks gestation, and then both groups were supplied with routine iron (60 mg) and folic acid 250 μg) supplements and anti-malarial prophylaxis. Serial ultrasound scans were performed and newborn anthropometry was recorded. Primary outcomes were mid-gestation indices of utero-placental vascular-endothelial function (ratio of plasminogen-activator inhibitor [PAI] 1 to PAI-2), mean uterine-artery resistance index and fetal-to-maternal measles antibody ratio as an index of placental active transport capacity at delivery.

Of 1156 women recruited, 376 had live singleton deliveries. There were no differences in PAI-1/PAI-2 or measles antibody ratio between trial arms, but there was a 0.02-unit reduction in uterine artery resistance index between 18 and 32 weeks of gestation (95% CI: -0.03, −0.00; *P* = 0.040) among women in the intervention group [2]. There was no significant effect of supplementation on birth weight. Two small pilot studies subsequently identified preliminary evidence of intervention-associated methylation differences in cord blood DNA, and in peripheral blood at the age of 9 months [24, 25]; no analyses were conducted relating DNA methylation to phenotypes.

For EMPHASIS, we aimed to study as many as possible of the 376 children; 356 were re-traced using the West Kiang Demographic Surveillance System [26], 298 of these were studied during 2016–2017 at the age of 7–9 years (Fig. 2). Similar outcomes were measured as in MMNP, using harmonised methods (Table 2). Blood samples and buccal swabs were collected for DNA and RNA and stored at -80 °C; DNA was isolated in The Gambia and DNA samples were transported to the laboratory in India on dry ice.

### DNA methylation profiling

DNA methylation profiling for both cohorts will be carried out at the CSIR-Centre for Cellular and Molecular Biology, Hyderabad, India. In a stage 1 ‘discovery’ analysis, genome-wide DNA methylation will be measured at >850,000 CpG methylation sites in ~700 Mumbai children and all the Gambian children with available DNA (*N* = 293) using the Illumina Infinium MethylationEPIC array (EPIC) (Illumina Inc., San Diego, USA), to identify differentially methylated positions (DMPs), regions (DMRs), and variably methylated positions and regions (VMPs and VMRs). Technical validation of significant DMPs and DMRs will be performed by pyrosequencing a subset of samples spanning the range of observed methylation values, using a Pyromark 96 pyrosequencer (Qiagen, Hilden, Germany). In addition, a small number of selected candidate loci not present on the EPIC array will also be assayed in both cohorts by pyrosequencing. These have been selected a priori following a literature review (manuscript in preparation) of other studies in which DNA methylation has been associated with maternal nutritional exposures and/or health outcomes of interest (Table 3). Replication of the technically validated loci will be performed using pyrosequencing in an independent sample of size *n* = 200–400 MMNP samples (sample size will depend on the observed effect size in the discovery analysis). In a cross-tissue analysis, technically validated significant loci will be examined in buccal DNA samples (n~50 from each cohort). All samples from both cohorts will also be genotyped using the Illumina Global Screening Array (GSA). Blood samples collected into Paxgene tubes for RNA isolation will be stored for later transcriptomic studies.

**Table 3:** Genes not on the EPIC array with previous evidence of associations with nutritional exposures and/or phenotypes

Locus	Genomic location	Associated exposures / outcomes	Refs
PAX8	chr2:113,992,866–113,993,036	Peri-conceptional nutrition exposure	[15]
POMC	chr2:25,384,508–25,384,832	Peri-conceptional nutrition exposure + phenotypic effect	[19]
HES1	chr3:193,849,141–193,849,361	Phenotypic effect	[42]
PPARGC1A	chr4: 23,892,404-23,892,571	Maternal BMI exposure	[13]
RBM46	chr4:155,702,818–155,703,110	Peri-conceptional nutrition exposure	[16]
NOS3	chr7:150,684,570–150,684,745	Phenotypic effect	[12, 43]
VIPR2	chr7:158,905,218–158,905,477	Famine exposure + phenotypic effect	[44, 45]
RXRA	chr9:137,215,689–137,215,826; chr9:137,215,979–137,216,126	Late gestation nutrition exposure + phenotypic effect	[12, 46]
H19	chr11:2,024,197–2,024,341	Peri-conceptional nutrition exposure	[47]
IGF2	chr11:2,169,457–2,169,541; chr11:2,169,617–2,169,751	Peri-conceptional nutrition exposure	[24, 48, 49]
MEG3 (GTL2)	chr14:101,294,220–101,294,391	Peri-conceptional nutrition exposure + phenotypic effect	[50]

### Data analysis

A detailed analysis plan can be found on the EMPHASIS website (www.emphasisstudy.org).

**Fig. 3:**
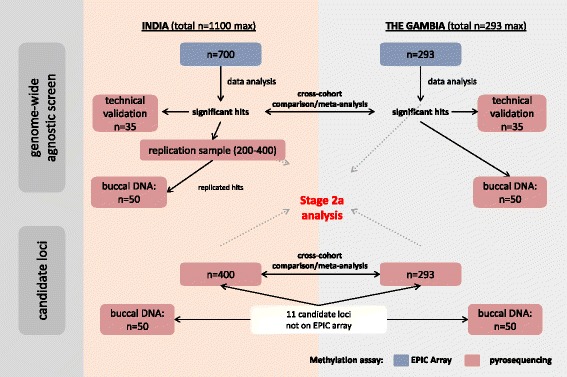
Stage 1 analysis of the impact of the nutritional interventions on DNA methylation

*Stage 1: Intervention-methylation associations (*Fig. 3 *):* Data from the two cohorts will be analysed separately. In a ‘hypothesis-free’, genome-wide analysis, the raw intensity data from the EPIC arrays will undergo pre-processing, quality control and normalization. Intervention-methylation associations will be identified at DMRs and DMPs using appropriate methods, and controlling for the false discovery rate (FDR). Loci and regions showing differences in methylation variance (VMPs and VMRs) will be identified, both genome wide and in an analysis targeted to MEs and imprinting control regions (ICRs). The candidate gene data will be analysed in parallel, using a similar strategy to the one outlined above to identify methylation differences associated with nutritional intervention. Technical validation will be carried out in a sub-set (10%) of samples using pyrosequencing. Significant hits will be those with *p* value <0.05 after correction for multiple testing. For the cross-tissue analysis, correlations of blood versus buccal methylation will be assessed using Pearson correlations.

Statistical power is estimated based on a sample size of 700 in the Indian cohort and 293 in The Gambia, to detect DMPs at a single locus using two-sample t-tests with alpha = 0.05, using a conservative assumption that tested loci have a methylation standard deviation at the 95th percentile of those observed (ie. within the 5% most variable probes). Significance thresholds were Bonferroni-adjusted assuming 800,000 independent tests, allowing for some filtering of probes. We estimate that we will be able to detect mean methylation differences between intervention and control groups, with 80% power, of 3 and 5% in the Indian and Gambian cohorts respectively. For the replication study using pyrosequencing in independent samples from the Indian cohort, and in the candidate loci studies in both cohorts, we estimate 80% power to detect a 2% difference in the Indian cohort and 3% in the Gambian cohort.

**Fig. 4:**
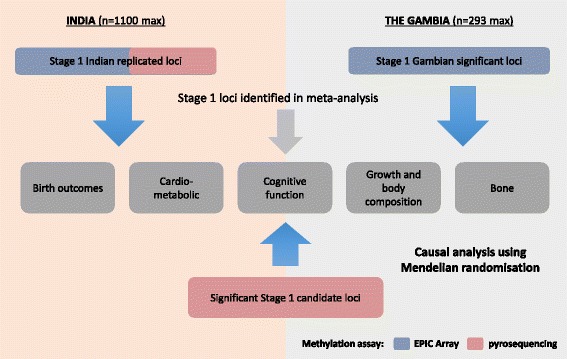
Stage 2 Associations of intervention-associated DMRs and loci with health outcomes

**Fig. 5:**
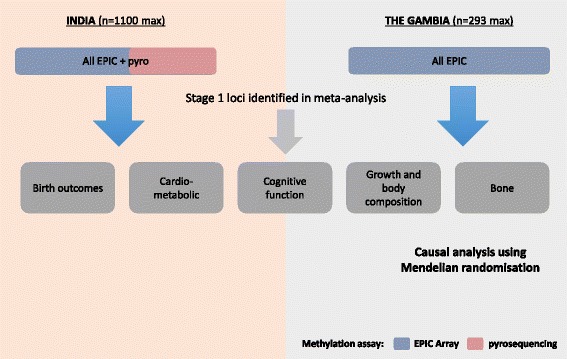
Associations of methylation and outcomes

*Stage 2: Methylation-outcome associations (*Figs. 4 and 5 *,* Table 4 *):* Significant loci associated with the nutritional intervention in either cohort from the EPIC array analysis, and all candidate loci, will be tested for associations with phenotype data measured in the children at the time of DNA collection, and also with birth outcomes (newborn anthropometry and gestation) (Table 4, Fig. 4). Loci identified in a separate meta-analysis of Stage 1 associations across both cohorts will also be considered. We will additionally carry out a ‘hypothesis-free’ analysis to identify loci where methylation is associated with outcomes, irrespective of intervention-methylation associations (Fig. 5).

**Table 4:** Phenotypic outcomes in the children in both cohorts

Domain	Primary outcomes	Secondary outcomes
Birth outcomes	Measured:	Measured:
	Birth weight (g) Birth length (cm)	Head, chest, abdomen and mid-upper arm circumferences (cm) Triceps and subscapular skinfolds (mm)
	Derived**:** Small for gestational age (SGA, N [%])^a^	Derived**:** Gestational age (weeks) Low birth weight (<2500 g) (N [%]) Pre-term (<37 completed weeks’ gestation) N [%])
At follow-up in childhood		
Anthropometry	Measured**:**	Measured**:**
	Standing height (cm)	Weight (kg) Sitting height (cm) Head, chest, waist, hip and mid-upper arm circumferences (cm) Biceps, triceps, subscapular, supra-iliac skinfolds (mm)
	Derived**:**	Derived**:**
	Body mass index (BMI) (kg/m^2^) Weight-, height- and BMI-for-age Z-scores^b^ (SD)	Stunted, wasted, underweight^b^ (N [%]) Leg length (cm) Sitting height/leg length ratio Head circumference-for-age Z-score^b^ (SD) Sum of skinfolds (mm) Waist/hip ratio Longitudinal growth measures
Body composition (DXA**)**	Measured:	Measured:
	Total lean mass (kg) Total fat mass (kg)	Android fat (kg) Gynoid fat (kg)
	Derived: Lean mass index (kg/m^2^) Fat mass index (kg/m^2^)	Derived: Body fat %
Bone (DXA and pQCT)	Measured:	Measured:
	*DXA:* Total and spine bone area (BA) (cm^2^) Total and spine bone mineral content (BMC) (g) Derived: *DXA:* Spine bone mineral apparent density (BMAD; (g/cm^3^)	*DXA:* Total and spine bone mineral density (BMD) (g/cm^2^) *pQCT (Gambia only):* Metaphyseal (8%) and diaphyseal (50%) tibia. Measurements taken using voxel size 0.5 mm, slice thickness 2 mm. Tibial total and trabecular BA (mm^2^) and volumetric BMD (vBMD) (mg/mm^3^). Diaphysial BA (mm^2^), BMC (mg/mm), vBMD (mg/mm^3^), cortical area (mm^2^) and thickness (mm), and strength (cross-sectional moment of inertia) (mm^4^).
Cardio-metabolic risk markers	Measured:	Measured:
	Systolic blood pressure (mmHg) Fasting glucose (mmol/l) 30- &120-min glucose (mmol/l) LDL-cholesterol (mmol/l) HDL-cholesterol (mmol/l) Triglycerides (mmol/l)	Diastolic blood pressure (mmHg) Fasting insulin (pmol/l) 30-min insulin (pmol/l)
	Derived:	Derived:
	Insulin resistance (HOMA-IR)^c^ Disposition index^d^	High blood pressure (mmHg)^e^ Insulinogenic index^f^ Metabolic syndrome N [%])^g^
Cognitive function	Measured:	
	Scores from Atlantis, Pattern reasoning, Kohs block design, Word order, Verbal fluency and Coding tests	
	Derived**:**	
	Mental processing index^h^ (SD)	

**Legend:** ^a^ SGA defined as below the 10th percentile for birth weight for gestational age using INTERGROWTH data [51]

^b^ according to WHO/CDC growth reference: http://www.who.int/growthref/en/

^c^ Insulin resistance according to Homeostasis Model Assessment: https://www.dtu.ox.ac.uk/homacalculator/

^d^ Disposition index: an estimate of insulin secretion taking into account insulin resistance, to be calculated as insulinogenic index/HOMA-IR [52]

^e^ High blood pressure defined as >99th percentile according to an international reference: https://www.nhlbi.nih.gov/health-pro/guidelines/current/hypertension-pediatric-jnc-4/blood-pressure-tables

^f^ Insulinogenic index: an estimate of first-phase insulin secretion, calculated as (insulin at 30 min – fasting insulin)/(glucose at 30 min – fasting glucose) [53]

^g^ Metabolic Syndrome: There is no accepted definition of metabolic syndrome in children of this age; a binary variable will be created, where 1 represents children who are above the highest sex-specific within-cohort quartiles for android fat on DXA, systolic blood pressure, plasma triglyceride concentration and HOMA-IR, and below the lower quartile for HDL-cholesterol

^h^ a composite score of cognitive function, calculated as the mean of the standardised scores from the 6 individual cognitive tests

#### Other analyses

To gain insights into underlying mechanisms, gene pathways analysis will be performed for the intervention-methylation-outcome genome wide association analysis. We will compare results from the intervention-methylation, methylation-outcome, and pathways analyses between the cohorts to identify commonalities and differences and explore further opportunities for meta-analysis. We will examine potential single nucleotide polymorphism (SNP) effects on methylation through methylation Quantitative Trait Loci (mQTL) analysis using the genome-wide genotype data on the children. We will also consider options for performing causal analysis with generated genotype data using Mendelian Randomisation (MR) [27–29].

DNA methylation assays for the discovery sample of Mumbai children and all of the Gambian children are scheduled to be completed in late 2017. Assays for the replication sample of Mumbai children will be completed in early 2018. The full EMPHASIS analysis (Stages 1 and 2) will be completed by mid-late 2018.

## Discussion

EMPHASIS is the first study in humans to examine the effects of maternal pre- and peri-conceptional nutrition on genome wide DNA methylation in children in a randomized controlled trial setting and to relate nutrition-related DNA methylation to a range of health outcomes. Recent technical advances offer the ability to study the methylome at high resolution and affordable cost. This gives us an unprecedented opportunity to investigate the effects of nutrition on methylation at a critical period (peri-conception), when the epigenome undergoes extensive remodelling. Most previous studies investigating these effects in humans have been observational, with limited scope for causal inference due to issues of confounding and reverse causality, or are quasi-experimental (eg famine studies) with imprecise exposure measures and/or large losses to follow-up. EMPHASIS is a unique opportunity to test the developmental origins of health and disease (DOHaD) hypothesis [5, 6, 9] and its underlying mechanisms.

The two-country design has strong advantages. Findings replicated in both cohorts will provide persuasive evidence for globally-relevant mechanisms with implications for policy. Differences between the cohorts will delineate some of the complex interrelationships between ethnicity, environment, nutrition and epigenetics, highlighting important context-specific factors. The two trials have commonalities and differences. The intervention comprised multiple micronutrients in both (from foods in MMNP and tablets in PMMST); the quantities of micronutrients were about fourfold higher in PMMST, while the MMNP food-based supplements provided some nutrients not present in the tablets used in PMMST (e.g. fatty acids). The timing of the intervention differed (continued throughout pregnancy in MMNP, stopped in early pregnancy in PMMST). The baseline nutritional status of the populations differed (the mothers in India were thinner and shorter, and the babies more growth restricted, than in PMMST (Table 2)). Vitamin B12 deficiency is common in India but not in The Gambia; and seasonal variation in diets is more marked in West Kiang than in Mumbai. We therefore expect the findings to reflect these commonalities and differences, revealing both shared and cohort-specific effects.

Evidence of cross-tissue, genotype-independent stochastic variation in DNA methylation at nutrition-associated loci will provide strong evidence that these loci are MEs, programmed in the early embryo. Evidence of nutrition-related epigenetic programming at peri-conception with the potential to influence gene expression in multiple tissue types would be an important finding. Methylation data from two tissues of different developmental origin will give further information about their stability across populations. The potential utility of buccal cells to measure epigenetic changes will in future allow non-invasive testing at multiple points in the lifecourse and relationships with disease progression to be followed.

High resolution genomic data will enable the investigation of potential confounding effects due to mQTL, genetic variants that influence methylation [30–34]. This may be particularly relevant for cross-cohort replication where differences in genetic background between cohorts should be taken into account. The mQTL can also be used as genetic instruments for causal analysis using MR [27–29]. Our study design is particularly well suited for ‘two-sample’ MR where instruments (mQTL) are identified in one sample, and analysed for their association with phenotype in the other. A related and particularly powerful approach is to use existing large genome wide association study (GWAS) datasets with relevant phenotypes and a similar genetic background as the second cohort in a two-sample MR analysis.

A limitation of EMPHASIS is the relatively small size of the Gambian sample, reducing the power to detect small methylation changes. The lack of perinatal DNA samples, in both cohorts, for methylation assays makes it more difficult to establish evidence for the direction of causality when correlating methylation with birth outcomes, although causal analysis would be expected to help to in this respect.

South Asian and sub-Saharan African countries stand out on the world map of maternal undernutrition and micronutrient deficiencies, low birth weight and childhood stunting [35]. Despite large investment in supplementation programmes for pregnant women there has been slow progress in reducing intra-uterine growth restriction and stunting. The evidence that adult non-communicable disease (NCD) risk is increased by fetal and infant under-nutrition suggests that these persistent problems contribute to the high and rising burden of NCDs in these countries [36, 37]. EMPHASIS will improve understanding of the biology linking maternal nutrition to fetal development and later health, potentially leading to better interventions.

## Abbreviations

BA: Bone area
BMAD: Bone mineral apparent density
BMC: Bone mineral content
BMD: Bone mineral density
BMI: Body mass index
CDC: Centers for disease control and prevention
CpG: Cytosine (phosphate) guanine di-nucleotide
CSIR: Council of Scientific and Industrial Research, India
DMP: Differentially methylated position
DMR: Differentially methylated region
DOHaD: Developmental origins of health and disease
DXA: Dual-energy X-ray absorptiometry
EMPHASIS: Epigenetic mechanisms linking pre-conceptional nutrition and health assessed in india and Sub-Saharan Africa
EPIC: Illumina Infinium methylationEPIC beadChip
FDR: False discovery rate
GSA: Illumina global screening array
GWAS: Genome wide association study
HDL: High density lipoprotein
HOMA-IR: Homeostasis model assessment – insulin resistance
ICR: Imprinting control region
LDL: Low density lipoprotein
LMICs: Low- and middle-income countries
ME: Metastable epiallele
MMNP: Mumbai maternal nutrition project (Mumbai, India)
mQTL: Methylation quantitative trait loci
MR: Mendelian randomization
NCD: Non-communicable chronic disease
PAI: Plasminogen activator inhibitor
PMMST: Peri-conceptional multiple micronutrient supplementation trial (The Gambia)
pQCT: Peripheral quantitative computed tomography
RNA: Ribonucleic acid
RNI: Reference nutrient intake, sufficient to meet the needs of >97% of people
SGA: Small for gestational age
SNP: Single nucleotide polymorphism
UNIMMAP: United nations multiple micronutrient preparation
vBMD: Volumetric bone mineral density
VMP: Variably methylated position (differential variance in methylation)
VMR: Variably methylated region (differential variance in methylation)
WHO: World Health Organization
